# The Implications and Predictability of Sleep Reversal for People with Myalgic Encephalomyelitis/Chronic Fatigue Syndrome: A Machine Learning Approach

**DOI:** 10.3390/healthcare13111255

**Published:** 2025-05-26

**Authors:** Meghan P. Dietrich, Raam Pravin, Jacob Furst, Leonard A. Jason

**Affiliations:** Center for Community Research, DePaul University, 990 W Fullerton Ave, Ste 3100, Chicago, IL 60614, USA; jfurst@cdm.depaul.edu (J.F.); ljason@depaul.edu (L.A.J.)

**Keywords:** ME/CFS, chronic illness, sleep dysfunction, functional impairment, sleep reversal, random forest, circadian rhythm disruption

## Abstract

Background/Objectives: Impaired sleep is one of the core symptoms of myalgic encephalomyelitis/chronic fatigue syndrome (ME/CFS), yet the mechanisms and impact of sleep-related issues are poorly understood. Sleep dysfunctions for patients with ME/CFS include frequent napping, difficulties falling asleep, waking up early, and sleep reversal patterns (e.g., sleeping throughout the day and staying awake throughout the night). The current study focuses on sleep reversal for patients with ME/CFS. Methods: We explored the symptoms and functional impairment of those with and without sleep reversal by analyzing the responses of a large international sample (N = 2313) using the DePaul Symptom Questionnaire (DSQ) and Medical Outcomes Study 36-item Short-Form Health Survey (SF-36). Results: We found that those in our Sleep Reversal group (N = 327) compared to those without sleep reversal (N = 1986) reported higher symptom burden for 53 out of 54 DSQ symptoms and greater impairments for all six SF-36 subscales. The most accurate predictors of sleep reversal included age (*p* < 0.05), body mass index (*p* < 0.05), eleven DSQ symptoms (*p* < 0.01), and two SF-36 subscales (*p* < 0.01). Conclusions: These features provide clues regarding some of the possible pathophysiological underpinnings of sleep reversal among those with ME/CFS.

## 1. Introduction

Myalgic encephalomyelitis/chronic fatigue syndrome (ME/CFS) is a chronic illness with key symptoms including (but not limited to) unrefreshing sleep, post-exertional malaise, and cognitive impairment [1,2,3]. Sleep difficulties are some of the most common symptoms experienced by patients with ME/CFS [4,5]. There are different subtypes of sleep disorders for those with ME/CFS [3,4,6], and suboptimal sleep quality is related to more frequent and severe ME/CFS symptoms [7]. Regarding patients’ self-reported sleep difficulties, Jason and Sunnquist [8,9] found that among patients with ME/CFS, 76% had unrefreshing sleep, 46% had problems falling asleep, 55% dealt with excessive daytime napping, 37% had problems staying asleep, 32% woke up very early in the morning, and 10% slept during the day and stayed awake at night (sleep reversal).

Sleep dysfunction is defined differently by CFS, ME, and ME/CFS case definitions. For example, the Fukuda et al. 1994 [10] CFS criteria specify “unrefreshing sleep” as one of eight key symptoms for diagnosis (requiring patients to meet at least four of the eight), but their requirements do not include other forms of sleep impairments. The Canadian Consensus Criteria [1] highlights sleep disturbance as a key facet of ME/CFS and includes other sleep disorders such as irregular/disruptive sleep rhythms, insomnias, hypersomnia, sleep reversal, or chaotic diurnal rhythms. The ME International Consensus Criteria [2] identifies unrefreshing sleep and disturbed sleep patterns as two separate categories of sleep dysfunction. According to the International Consensus Criteria [2], examples of sleep disturbances include insomnia, hypersomnia, frequent napping, sleep reversal patterns, repeated awakenings throughout the night, abnormally early awakening in the morning, and vivid dreams or nightmares. Sleep reversal is recognized by the International Consensus Criteria as notable in the chronic stage of ME/CFS. Unrefreshing sleep is a core symptom according to the Institute of Medicine [3] criteria, but similar to the Fukuda [10] criteria, no other sleep-related issues are required to meet this ME/CFS diagnostic criteria.

Several ME/CFS studies have used objective measures to assess sleep impairment [4,5,6]. Maksoud et al. [4] reviewed such studies that included polysomnography, apnea–hypnea index, microarousal index levels, and multiple sleep latency testing measures. Five studies found that microarousal index levels were higher for those with ME/CFS than healthy controls. McCarthy et al.’s review [11] found that genetic variation in clock genes may lead to dysregulated sleep patterns among patients with ME/CFS. They also found that the symptoms of dysautonomia, including abnormal body temperatures, irregular heart rate, and blood pressure, and altered cortisol levels, are linked to ME/CFS circadian rhythm irregularities [11].

One of the aforementioned sleep dysfunctions for those with ME/CFS is sleep reversal (or sleep inversion), defined as the experience of sleeping during the day and staying awake at night. ME/CFS factor analytic studies often remove this symptom from their analyses due to its low factor loading [12,13]. Sleep reversal is a form of circadian rhythm dysfunction, and it is generally seen in the form of shift work syndrome or jet lag [11,14,15]. Such rhythm disturbances are thought to impair physical functionality and lead to autonomic, cognitive, and fatigue-related problems. Sleep reversal patterns are also prominent in two other circadian rhythm sleep disorders: delayed sleep phase syndrome and insomnia [16]. Those with delayed sleep phase syndrome experience an inability to fall asleep at early, regular hours. They consistently fall asleep late at night or early in the morning, shortly before an ideal awakening time, and wake up in the late morning or afternoon. Although their sleep schedule is shifted, their sleep quality tends to be rather normal. For those with insomnia, sleep onset latency and sleep quality are severely impaired. Insomnia leads to trouble staying asleep and frequent awakenings throughout the night, thus causing a lower overall sleep quantity [16].

Although sleep reversal has been studied in several circadian rhythm sleep disorders [11,14,15,16], it has not been made a focus within the ME/CFS literature. The current study aimed to explore the differences in symptom manifestation between those with ME/CFS who report experiencing sleep reversal patterns and those who do not report this problem. We hypothesized that those with sleep reversal would have more overall symptoms, measured by the DePaul Symptom Questionnaire, and higher levels of impaired functioning, measured by the Medical Outcomes Study (MOS) Short-Form Health Survey.

## 2. Methods

This study utilized an international aggregate dataset of 2313 participants with ME/CFS. The aggregate is composed of data from ten independent samples from six countries. Conroy et al. [12] provide a detailed breakdown of each sample, which we briefly summarize below.

### 2.1. DePaul Sample

An international convenience sample of 214 adults with ME/CFS ages 19–79 years (M = 52.0, SD = 11.3) were recruited by DePaul University investigators. The majority of participants were White (97.2%), female (83.6%), and on disability (57.0%).

### 2.2. Solve ME/CFS BioBank Sample

A sample of 502 adults ages 18–88 years (M = 54.8, SD = 12.0) were recruited through Solve ME/CFS’ website, Solve ME/CFS’ social media accounts, and physician referral. Most of the participants with ME/CFS were White (93.8%), female (74.21%), and nearly half were on disability (44.8%).

### 2.3. Newcastle Sample

This sample included 95 adults diagnosed by a physician experienced in ME/CFS diagnosis after being referred due to a suspected diagnosis of CFS. Ages ranged from 19 to 74 years (M = 45.8, SD = 14.1), and participants were mainly White (98.9%), female (82.1%), and about a third were on disability (30.5%).

### 2.4. Norway 1 Sample

This sample of 173 adults aged 18–78 years (M = 43.3, SD = 11.7) was diagnosed with CFS by a physician or medical specialist, and recruited from Oslo and nearby communities. The majority of participants were White (99.4%), female (86.7%), and on disability (83.8%).

### 2.5. Norway 2 Sample

A sample of 60 adults ages 18–65 years (M = 35.4, SD = 11.7) were recruited from an outpatient clinic at a multidisciplinary ME/CFS Center and an inpatient ward for extremely ill patients. Most participants were White (95.0%), female (81.7%), and on disability (81.7%).

### 2.6. Norway 3 Sample

This sample of 169 adults met the Canadian Consensus Criteria for ME/CFS from Oslo University Hospital. Their age ranged from 18 to 63 years (M = 38.6, SD = 11.3). Participants were mostly White (96.4%), female (81.7%), and on disability (91.1%).

### 2.7. Chronic Illness Sample

A convenience sample of 435 adults with ME/CFS, aged 19–79 years (M = 52.0, SD = 11.3), was recruited through social media outlets, research forums, national foundations, and support group websites. Participants were primarily White (96.32%), female (88.7%), and about half were on disability (48.7%).

### 2.8. Japanese Sample

This sample of 124 adults was recruited through the ME Japan Association and its ME/CFS-specializing clinics. The sample’s ages ranged from 20 to 82 years (M = 46.1, SD = 13.5). All participants were Asian, mostly female (78.2%), and a quarter were on disability (25.0%).

### 2.9. Spain Sample

This sample of 183 adults met the Fukuda et al. [10] case definition for ME/CFS and were recruited from a tertiary referral center located in Barcelona. The sample age ranged from 21 to 74 years (M = 50.4, SD = 8.6). The majority of participants were White (99.5%), and female (85.2%), and more than a third of them were on disability (39.9%).

### 2.10. Netherlands Sample

This sample of 358 adults was recruited from the CFS Medical Center’s outpatient clinic in Amsterdam, Netherlands. Their ages ranged from 18 to 72 years (M = 37.1, SD = 11.5). Most of the participants were female (77.9%), and more than a third had a college degree, master’s degree, or doctorate (42.0%).

## 3. Measures

### 3.1. The DePaul Symptom Questionnaire

All participants filled out the DePaul Symptom Questionnaire (DSQ). The DSQ is a survey measuring the frequency and severity of 54 symptoms experienced over the last six months. The frequency of a symptom was measured on a 5-point Likert scale (0 = none of the time, 1 = a little of the time, 2 = about half the time, 3 = most of the time, 4 = all of the time), and the severity was also measured on a 5-point scale (0 = symptom not present, 1 = mild, 2 = moderate, 3 = severe, 4 = very severe). Composite scores were calculated for each DSQ symptom item by averaging the corresponding frequency and severity scores together, then multiplying by 25 to create a 100-point scale, with higher scores representing a larger burden to patients. The 54 symptoms included in this survey represent symptom domains outlined in the Canadian Consensus Criteria (CCC) [9]. An individual was considered to meet symptom criteria if they had a composite score of at least 50 points for a given symptom. The DSQ also collects demographic information from respondents, including height, weight, gender, race, ethnicity, marital status, and work status. The DSQ has demonstrated good-to-excellent test–retest reliability [17], as well as strong internal consistency [8].

### 3.2. The Medical Outcomes Study (MOS) Short-Form Health Survey (SF-36)

Participants also completed the MOS SF-36. The 36 items included in this survey contain 7 binary yes/no questions, and 29 3-to-6-point Likert scale questions that evaluate participants’ health across the following eight subscales: physical functioning (ten items); role limitations due to physical health problems (four items); bodily pain (two items); general health functioning (five items); vitality (four items); role limitations due to personal or emotional problems (three items); social role functioning (two items); and mental health (five items). Each item response is converted to a 100-point scale. The SF-36 is not used to diagnose ME/CFS but is rather used as a tool to gain further insight regarding patients’ daily health experiences, including how their symptoms impact varying facets of their lives. Higher scores indicate better functioning. Among those with ME/CFS and other patient groups, the SF-36 has exhibited strong internal consistency and reliability [18].

### 3.3. Missing Values

Analyses were performed in R (version 4.3.1) in RStudio (version 2023.12). Participants were only included in the analysis if they completed at least 90% of the 54 DSQ symptom items. Of the 2402 participants, 96.3% (n = 2313) were successfully retained. Less than 5% of the data values were missing from these remaining participants. Missing values were entered by adopting a previously used approach [12,19,20,21]. For instances where there was a score of 0 for only the frequency or severity of a symptom, a 0 was input for the corresponding missing value. The reasoning is that a symptom can only occur “none of the time” (frequency = 0) when the symptom is “not present” (severity = 0). In all other instances where one of the two fields (frequency or severity) was missing a value, the mode from the cases that had the corresponding score was entered. In instances where both fields were missing values, the corresponding symptom frequency and severity medians across the sample were entered. Basic descriptives revealed that six cases of incorrect data existed for the BMI variable (BMIs lower than 5, and greater than 100). After the imputation of null values in these instances, 99.7% of the BMI values in the dataset were still retained.

### 3.4. Random Forest Algorithm

The random forest algorithm is a supervised machine learning algorithm commonly used for classification and regression [22]. The algorithm uses bootstrapping to sample the original dataset with replacement and create subsets. Each subset is the same size as the original dataset, and since the original dataset is sampled with replacement, there is a high probability that each subset contains different data points. For each subset, a decision tree is built by randomly selecting a subset of predictors from all available predictors. Each decision tree makes a decision or vote. For classification, the majority of votes are considered as the model’s prediction on a particular data point. For regression, the average of all votes is considered the model’s prediction on a particular data point. Because the random forest randomly selects a subset of features to use for each decision tree it builds, there is a low correlation among each decision tree. Additionally, low variance is ensured in classification since each tree is trained on a different subset of the training data, and the final prediction is made by taking the prediction that the majority of models voted for. This helps build robust models, which work well even on unseen data.

### 3.5. Selection of Important Features: Mean Decrease in the Gini Coefficient

Gini impurity is a measure used in decision trees that evaluates the chance a randomly selected element from the subset would be incorrectly labeled. To evaluate feature importance in the random forest models, we used the mean decrease in Gini coefficient, which is calculated by averaging the decrease in Gini impurity caused by splitting on the feature in every node and tree in the forest. The higher the average value is, the more important the feature is in the random forest.

### 3.6. Determining a Threshold

Whether or not a participant experienced sleep reversal was determined based on both the frequency and severity scores for the DSQ item “Sleep all day and stay awake all night”. The sleep reversal threshold has been defined as a frequency score of 2 (about half the time) or greater, and a severity score of 2 (moderate) or greater. This excluded participants who reported a 0 (none of the time) or 1 (a little of the time) for frequency, or those who reported a 0 (symptom not present) or 1 (mild) for the severity of this symptom. Those who experience a symptom mildly and/or a little of the time are not necessarily burdened or impacted by the symptom in their daily lives, as opposed to those who experience a symptom at least moderately and about half the time or more [8]. The Sleep Reversal group contained 14.1% (N = 327) of the sample, while the No Sleep Reversal group contained 85.9% (N = 1986) of the sample.

### 3.7. Exploring Group Differences

We compared the demographic characteristics of those with and without sleep reversal. An ANOVA (analysis of variance) and Chi-square tests were conducted to investigate if there were significant demographic differences between the two groups. If at least one-third of the DSQ or SF-36 items were found to be affected by a covariate, we then adjusted for the covariate by calculating and using the estimated marginal means instead of the raw means. The estimated marginal means sets covariates to a constant and calculates a new mean, allowing for the determination of the true difference between groups [23].

ANCOVAs (analysis of covariance) and Chi-square tests evaluated the differences between those with and without sleep reversal. A *p*-value of 0.00125 was chosen using Bonferroni correction, which corrects for increased error rates due to multiple comparisons [24].

### 3.8. Random Forest Predictive Model Selection

We investigated if sleep reversal incidence could be predicted. The data were partitioned on an 80/20 split for training and testing. Eight different random forest models were evaluated using the plotting of receiver operating characteristic (ROC) curves. According to Kumar and Indrayan [25], the ROC curve plots the exchange between the sensitivity (true positive rate) and specificity (true negative rate) at various thresholding values, and the AUC (area under the ROC curve) is utilized to measure the robustness of a specific model. The first model included all DSQ items, SF-36 items, and demographic characteristics. To evaluate variable importance in the random forest model, the mean decrease in Gini was used to rank the most important variables. The models evaluated included the 40, 35, 30, 25, 20, 15, and 10 most important variables, respectively. The VSURF (variable selection using random forests) method [26] was also utilized to create a predictive model to be evaluated. Because there was a substantial class imbalance in the two groups (only 14.4% of participants experienced sleep reversal), each model was trained on two datasets: the original dataset with the class imbalance, and a synthetically composed dataset which randomly sampled from the minority class with replacement. This eliminated the class imbalance between participants with sleep reversal and participants without sleep reversal. The produced random forest classification models were evaluated through the plotting of the ROC curve and the computation of the AUC. For each set of features, 31 iterations were run, thus creating 31 different random forest models, with the AUC recorded for each one. To evaluate the performance of these models and identify the best-performing one, a 95% confidence interval for the AUC was made to evaluate which model had the highest mean AUC and smallest confidence interval.

## 4. Results

Table 1 displays the sociodemographic characteristics of the Sleep Reversal and No Sleep Reversal groups. Both the Sleep Reversal and No Sleep Reversal groups were majority female and White. The following five demographic categories were significantly different between those with and without sleep reversal: BMI, age, marital status, and work status, Hispanic or Latino.

The Sleep Reversal group was significantly younger and had a higher average BMI. The Sleep Reversal group also had a smaller proportion of participants who were married or living with a partner, a slightly higher proportion of participants who were separated, widowed, or divorced, and a higher proportion of participants who never married compared to the No Sleep Reversal group. The Sleep Reversal group also had a smaller proportion of participants who were retired, working part-time, or working full-time, and a higher proportion of those who were unemployed than the No Sleep Reversal group. Of the 135 Hispanic/Latino participants, 116 were Hispanic from Spain, leaving only 19 participants of Latin American origin in this category. Due to this discrepancy, we did not adjust for the Hispanic/Latino variable in further analyses.

The following three demographic variables affected at least one-third of DSQ item scores: age, marital status (never married), and work status (disabled). The DSQ means were adjusted for when calculating the estimated marginal means. Table 2 shows the estimated marginal means of every DSQ symptom for the Sleep Reversal and No Sleep Reversal groups. Sensitivity to alcohol was the only symptom that was not significantly different between groups; all remaining 53 DSQ item composite score means were significantly higher in the Sleep Reversal group, indicating a higher level of overall symptomatology for this group of participants.

The following three demographic variables affected at least one-third of SF-36 item scores: age, work status (part/full-time), and work status (disabled). SF-36 item score means were adjusted for calculating the estimated marginal means. Table 3 displays the results of the SF-36 across the Sleep Reversal and No Sleep Reversal groups. Participants in the Sleep Reversal group scored worse than the No Sleep Reversal group in every domain. These findings indicate that members of the Sleep Reversal group experienced significantly more impairments than those in the No Sleep Reversal group.

### Random Forest Final Model

**Figure 1:**
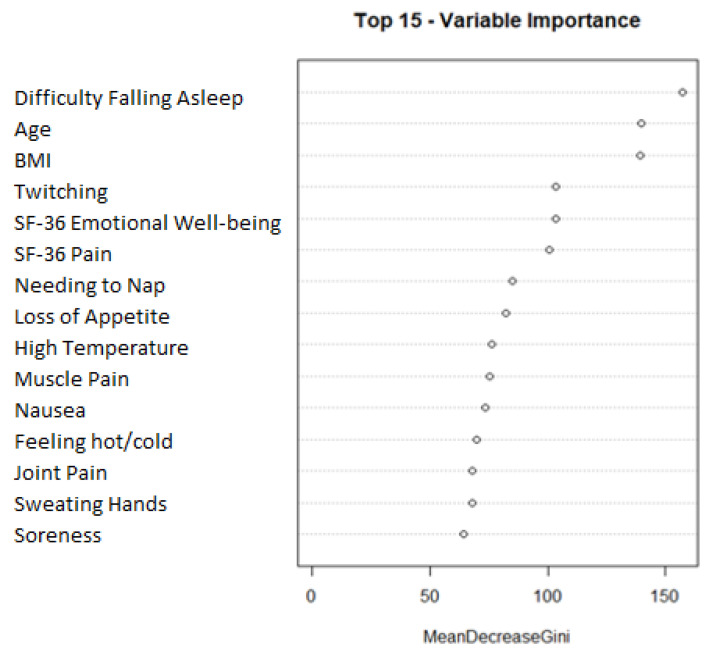
Feature importance plot for final random forest model. Final model variable importance ranked by mean decrease gini.

We next predicted sleep reversal using a random forest classification algorithm. The most robust model was made through oversampling and composed of the following 15 variables: difficulty falling asleep, age, BMI, twitching, SF-36 emotional well-being, SF-36 pain, needing to nap, loss of appetite, high temperature, muscle pain, nausea, feeling hot/cold, joint pain, sweating hands, and soreness. Figure 1 shows a plot visualizing the mean decrease Gini of the features in the final model. The mean decrease Gini plot shows that difficulty falling asleep, age, and BMI were the most prominent features in the random forest model. A 95% confidence interval of the AUC was built from the 31 iterations of the final model: (0.7643:0.7831).

The top 15 most useful variables in predicting sleep reversal are listed in order according to our final random forest model.

To represent the predictability of the final model, a 95% confidence interval was also built from the 31 iterations. A confusion matrix and performance measures of the iteration closest to the mean accuracy are shown in Table 4. The model’s overall accuracy was 74.46%, and 32 participants with sleep reversal were correctly classified as having sleep reversal, while 242 participants without sleep reversal were also correctly identified.

## 5. Discussion

Our study found that those with sleep reversal were more impaired than those without sleep reversal on almost all DSQ symptoms and all SF-36 subscale scores. These findings indicate that the sleep reversal symptom does identify a severely impaired subtype of patients with ME/CFS. The most accurate random forest model contained 15 of the most important features in predicting sleep reversal, which included age, BMI, 2 SF-36 subscales, and 11 DSQ items. These features provide clues that are explored below regarding some of the possible pathophysiological underpinnings of sleep reversal among those with ME/CFS.

Difficulty falling asleep was the strongest predictor of sleep reversal. Of course, if a person has sleep reversal, by definition difficulty falling asleep would be indicated. This probably explains why needing to nap was also a feature in our predictive model: the relationship between napping and falling asleep may be bidirectional, in that excessive daytime napping could lead to having more difficulties falling asleep at night.

Age was the second strongest predictor of sleep reversal. Nacul et al. [27] found that as patients progressed through longer periods of ME/CFS, pro-inflammatory cytokine levels decreased, and anti-inflammatory cytokine levels increased. Further, Milrad et al. [7] discovered a negative relationship between sleep quality and pro-inflammatory cytokine levels for women with ME/CFS, indicating that higher levels relate to poorer sleep quality. Older patients have likely been experiencing ME/CFS longer than most younger patients, thus it is possible that older patients tend to have lower levels of pro-inflammatory cytokines and higher levels of anti-inflammatory cytokines compared to younger patients, potentially causing younger patients to experience more acute inflammation. This difference in inflammatory cytokine levels may partially account for the younger average age of our Sleep Reversal group, as inflammation negatively impacts one’s ability to sleep well [7]. It may also be possible that light exposure and technology usage are contributing factors to the lower average age of our study’s sleep reversal group. Although this was not a variable included in the data collection for this study, prior research has discovered that in a healthy sample, cell phone usage, as well as the usage of other interactive technology, is far more common in people younger than 30 years old than those who are older. The use of such devices typically leads to increased sleep onset latency, problems falling asleep, and unrefreshing sleep [28]. This may be an important factor for future researchers to account for in regards to participants with ME/CFS.

Other variables that were key in predicting sleep reversal, such as BMI, also have connections with inflammation. Wang et al. [29] investigated the correlation between BMI and proinflammatory cytokine levels with hematopoietic stem cell mobilization triggered by granulocyte colony-stimulating factors. This group found that BMI and proinflammatory cytokine levels were positively correlated. This suggests that a higher BMI may indicate more inflammation and poorer sleep quality, making it a feature in predicting sleep reversal.

Additionally, four pain features (e.g., muscle pain, joint pain, soreness, and the SF-36 pain subscale) were among the most important features in predicting sleep reversal. Certainly, chronic pain can interfere with one’s ability to sleep. Pain is strongly associated with inflammation [30], and the disruption of diurnal cortisol secretion can influence inflammation [31]. Torres-Harding et al. [32] found that for those with ME/CFS, pain severity was positively correlated with atypical cortisol levels. Several other studies have found a relationship between hypocortisolism and sleep in patients with ME/CFS [33]. For example, Scott et al. [34] found that for those with ME/CFS, urinary-free cortisol excretion was significantly lower than for those without ME/CFS. Because chronic circadian rhythm misalignment significantly reduces cortisol levels [35], it is possible that lower cortisol levels are related to pain and sleep difficulties in our sample. This is just a speculation, as we did not collect samples of cortisol in our study.

Both loss of appetite and nausea also predicted sleep reversal in our model. Chen et al. [36] found that among patients with cancer, gastrointestinal inflammation was an essential mechanism for chemotherapy-induced nausea and vomiting. It is possible that gastrointestinal inflammation was more pronounced in our Sleep Reversal group.

Garami et al. [37] found that systemic inflammation is generally accompanied by regulated changes in body temperature, which could manifest in the form of fever or hypothermia. If our sample’s Sleep Reversal group had more inflammation than the No Sleep Reversal group, this might be a reason that 3 of the 15 most important features in predicting sleep reversal were associated with changes to body temperature, including having a high temperature, feeling hot or cold for no reason, and having sweating hands. It is also likely that those who have the sleep reversal symptom have poorer thermoregulation than those without this symptom, particularly in the form of increased internal body temperatures. Prior research has displayed a bidirectional relationship between thermoregulation and physiological sleep mechanisms, and increased heat exposure often relates to lower sleep quality and greater sleeplessness [38].

Twitching was another predictor in our model. Restless leg syndrome is a movement disorder that manifests through the involuntary movement of legs during inactivity caused by the twitching of muscles which, when experienced at night, can disturb patients’ sleep [39]. Pajediene et al. [40] found that among patients complaining of chronic fatigue, 41% had restless leg syndrome. Sleep disruption leading to poorer overall quality of sleep in patients with ME/CFS has been found to be associated with restless leg syndrome [41]. As with restless leg syndrome, twitching might exacerbate the ability to sleep at night.

Those with sleep reversal had significantly lower SF-36 emotional well-being scores. Individuals in the general population who obtain suboptimal sleep experience higher levels of depression and anxiety, as well as lower levels of psychosocial adjustment [42]. As sleep reversal is a severe form of sleep dysfunction, it could be associated with more emotional impairment.

There are several limitations in this study. Sleep reversal was defined solely on a single DSQ item, which leaves room for possible variations in interpretation amongst participants. More research with biological variables alongside self-report measures could help to confirm the possible pathophysiological pathways discussed above. Additionally, our sample was primarily composed of white female participants, which limits the generalizability of our findings.

Although this study measures many symptoms across several domains, there may be other contributing factors to sleep reversal patterns that were not included in our data collection. A small number of participants in this study reported medication usage; thus, we were unable to analyze whether or not this impacted sleep reversal patterns. Additionally, in the general population, later feeding patterns have been found to relate to circadian rhythm misalignment and higher BMI [43]. It has also been found that those with evening chronotypes, or those who tend to go to sleep later at night, tend to have increased health issues, including higher BMI [41]. As BMI was found to be an important predicting factor in the present study, future research should look further into how eating habits and varying chronotypes may or may not contribute to the relationship between ME/CFS and sleep reversal. It is notable that a study by Rahman et al. [44] found that patients with ME/CFS tend to report earlier chronotypes and lower sleep quality than their study’s control group, which contradicts the aforementioned findings within the general population; but, it is possible that the chronotypes of those within the subgroup of individuals with ME/CFS who experience acute sleep reversal problems differ from the broader population of individuals with ME/CFS. More research must be carried out to understand this relationship.

Some researchers may focus on circadian light hygiene in patients with ME/CFS. Although there is a clear gap in existing research when it comes to light hygiene and sleeping patterns in patients with ME/CFS, it has been found that night shift workers, who have sleep reversal due to their work schedules, are more prone to contracting COVID-19 than the general population. The COVID-19 pandemic has led to a significant number of people developing long-term symptoms which mirror ME/CFS. This is currently known as the post-acute sequelae of COVID (PASC), or “Long COVID”, and research on this topic is still developing [11]. However, given the fact that night shift workers, whose primary difference from the general population is their daily light exposure, are more susceptible to this illness which often leads to ME/CFS and/or Long COVID, it is important for researchers to account for light hygiene patterns when further studying sleep reversal in those with ME/CFS. Insufficient exposure to light may detrimentally impact the circadian rhythm alignment of this patient group [11].

## 6. Conclusions

To summarize, our study provides evidence that sleep reversal identifies a more impaired subgroup within the population of individuals with ME/CFS. Based on previous research findings that increased levels of pro-inflammatory cytokines contribute to more impaired sleep patterns [7], it is possible that significant inflammation may be an underlying cause of sleep reversal. Studies suggest that inflammation may relate to a majority of the predictive variables in our study. Additionally, as prior research indicates a relationship between low cortisol levels and circadian rhythm misalignment [33,34], lower cortisol levels in those with ME/CFS could also act as a contributing factor to sleep reversal patterns. Future research is needed to explore the biological impact sleep reversal has on those with ME/CFS, and to better understand the mechanisms surrounding this condition.

## Figures and Tables

**Table 1:** Sociodemographic information for sleep reversal and no sleep reversal groups.

		Sleep Reversal	No Sleep Reversal	*p*-Value
Demographic		(N = 327)	(N = 1986)	
		M (SD)	M (SD)	
Age (years)		43.7 (13.5)	47.4 (13.6)	<0.05
BMI		27.3 (8)	25.5 (5.9)	<0.05
				
		% (n)	% (n)	
Gender				0.27
	Male	15.5 (50)	18.2 (357)	
	Female	84.5 (272)	81.8 (1601)	
Race				0.71
	White/Caucasian	91.0 (254)	91.7 (1512)	
	Black/African American	0.0 (0)	0.1 (2)	
	Asian/Pacific Islander	7.2 (20)	6.8 (112)	
	American Indian/ Alaskan Native	0.0 (0)	0.1 (2)	
	Other	1.8 (5)	1.0 (17)	
				
Hispanic or Latino		12.9 (36)	6.0 (99)	<0.05
				
Marital Status				<0.05
	Married or living with partner	52.8 (141)	65.5 (1095)	
	Separated/Widowed/ Divorced	6.0 (16)	5.4 (90)	
	Never married	41.2 (110)	29.2 (488)	
Work Status				<0.05
	On disability	51.4 (72)	48.2 (408)	
	Student/Homemaker/ Retired	15.0 (21)	21.6 (183)	
	Unemployed	22.9 (32)	14.4 (122)	
	Working part-time/ Working full-time	10.7 (15)	15.7 (133)	
				
Education Level				0.21
	High school/GED or less	7.0 (15)	4.4 (67)	
	Partial college or specialized training	22.8 (49)	21.4 (327)	
	Standard college degree	44.7 (96)	50.6 (773)	
	Graduate or professional degree	25.6 (55)	23.7 (362)	

Significant differences in age and BMI between the two groups were determined using independent *t*-tests at the *p* < 0.05 level. Significant differences in all other demographic categories were determined using the Chi-square test of independence at the *p* < 0.05 level.

**Table 2:** Group DSQ item composite score means by symptom domain.

		Sleep Reversal	No Sleep Reversal	
Symptom Domain		(N = 327)	(N = 1986)	*p*-Value
	Symptom	M (SD)	M (SD)	
Sleep				
	Unrefreshing Sleep	86.9 (15.1)	74.7 (20.9)	<0.01
	Needing to Nap	69.2 (27.5)	53.3 (30.5)	<0.01
	Difficulty Falling Asleep	75.1 (26.3)	54.2 (30.7)	<0.01
	Difficulty Staying Asleep	69.4 (28.9)	54.4 (30.6)	<0.01
	Waking up Early	59.0 (33.2)	47.0 (31.6)	<0.01
	Sleep Reversal	66.3 (15.6)	9.04 (15.8)	<0.01
PEM				<0.01
	Heavy Feeling	75.9 (25.8)	65.6 (28.7)	<0.01
	Mental Fatigue	74.1 (22.3)	62.0 (25.3)	<0.01
	Minimum Exercise	82.2 (18.6)	71.4 (23.9)	<0.01
	Feeling Drained	77.4 (20.4)	67.2 (25.2)	<0.01
	Fatigue	86.0 (13.8)	76.9 (17.8)	<0.01
	Muscle Weakness	70.7 (25.8)	57.3 (29.1)	<0.01
Neurocognitive				<0.01
	Difficulty Remembering	72.1 (25.1)	59.5 (26.6)	<0.01
	Trouble Paying Attention	75.4 (24.4)	65.9 (25.8)	<0.01
	Trouble Forming Words	68.8 (24.6)	57.2 (26.1)	<0.01
	Difficulty Understanding	56.2 (26.4)	44.9 (27.1)	<0.01
	Difficulty Focusing	71.5 (25.0)	60.8 (28.4)	<0.01
	Slowness of Thought	64.8 (26.6)	54.2 (27.3)	<0.01
	Sensitivity to Noise	65.1 (28.4)	53.2 (29.5)	<0.01
	Sensitivity to Light	63.4 (27.9)	48.7 (31.1)	<0.01
	Sensitivity to Smells	55.3 (34.2)	42.2 (34.0)	<0.01
	Unable to Focus Vision	59.4 (28.1)	46.0 (27.6)	<0.01
	Loss of Depth Perception	33.2 (31.9)	21.1 (28.1)	<0.01
	Twitching	50.7 (29.9)	31.3 (25.8)	<0.01
	Absent Mindedness	67.9 (26.3)	54.4 (27.2)	<0.01
Immune				<0.01
	Sore Throats	47.3 (26.9)	35.4 (26.1)	<0.01
	Lymph Nodes	45.5 (30.4)	33.7 (28.7)	<0.01
	Fever	27.3 (26.6)	17.6 (22.5)	<0.01
	High Temperature	45.8 (29.8)	28.1 (28.0)	<0.01
	Flu	56.1 (27.7)	44.6 (28.1)	<0.01
Neuroendocrine				<0.01
	Cold Limbs	59.1 (29.5)	49.7 (30.4)	<0.01
	Chills	43.8 (27.6)	31.7 (27.1)	<0.01
	Feeling Hot/Cold	62.2 (28.0)	43.7 (28.4)	<0.01
	Night Sweats	47.9 (29.8)	30.6 (28.8)	<0.01
	Sweating Hands	29.5 (31.2)	12.4 (22.8)	<0.01
	Weight Change	49.4 (35.1)	32.3 (33.6)	<0.01
	Loss of Appetite	39.4 (27.4)	23.2 (24.9)	<0.01
	Low Temperature	36.9 (30.5)	25.3 (28.4)	<0.01
Pain				<0.01
	Muscle Pain	75.8 (24.1)	62.9 (27.6)	<0.01
	Headaches	57.5 (25.6)	47.5 (26.4)	<0.01
	Eye Pain	45.1 (29.1)	30.0 (28.1)	<0.01
	Soreness	80.7 (17.9)	69.5 (23.4)	<0.01
	Joint Pain	70.2 (28.3)	52.4 (32.0)	<0.01
Gastrointestinal				<0.01
	Bloating	52.0 (29.7)	39.2 (28.9)	<0.01
	Bladder Issues	44.2 (33.5)	30.6 (31.8)	<0.01
	Sensitivity to Alcohol	39.6 (37.6)	36.3 (36.3)	0.22
	Stomach Pain	50.2 (27.9)	37.3 (28.2)	<0.01
	Irregular Bowels	52.6 (33.3)	41.7 (33.3)	<0.01
Orthostatic				<0.01
	Nausea	38.8 (26.4)	29.0 (25.8)	<0.01
	Chest Pain	38.1 (27.9)	24.1 (25.1)	<0.01
	Feeling Unsteady	57.9 (29.8)	40.2 (27.9)	<0.01
	Shortness of Breath	50.2 (29.0)	35.7 (27.7)	<0.01
	Dizziness	47.3 (27.5)	34.9 (26.4)	<0.01

DSQ item composite scores for all symptom domains were determined to be significantly higher in the Sleep Reversal group than the No Sleep Reversal Group (except for the symptom of alcohol sensitivity) using two-sample independent *t*-tests at the *p* < 0.01 level.

**Table 3:** Group SF-36 score means by subscale.

	Sleep Reversal	No Sleep Reversal	
Subscale	(N = 274)	(N = 1782)	*p*-Value
	M (SD)	M (SD)	
Physical functioning	27.7 (22.8)	35.5 (23.6)	<0.01
Role limitations due to physical health	4.6 (11.1)	7.01 (16.7)	<0.01
Role limitations due to emotional problems	50.9 (46.6)	61.7 (42.8)	<0.01
Energy/fatigue	14.2 (13.8)	15.8 (15.3)	<0.01
Emotional well-being	58.3 (22.2)	65.0 (19.2)	<0.01
Social functioning	21.8 (22.5)	29.6 (24.0)	<0.01
Pain	29.5 (24.2)	44.0 (26.4)	<0.01
General health	22.2 (14.8)	26.2 (16.1)	<0.01

Significant differences in symptoms between the two groups were determined using two-sample *t*-tests at the *p* < 0.01 level.

**Table 4:** Confusion matrix predicting sleep reversal.

Predicted Class	Actual Class		Sensitivity	Specificity
	No Sleep Reversal	Sleep Reversal		
**No Sleep Reversal**	242	11	74.42%	74.46%
**Sleep Reversal**	83	32		
	Accuracy: 74.46%			

**Iteration whose accuracy was closest to the mean**.

## Data Availability

The data presented in this study are available on request from author Dr. Leonard A. Jason (ljason@depaul.edu).

## Abbreviations

ANCOVA	Analysis of covariance
ANOVA	Analysis of variance
AUC	Area under the ROC curve
BMI	Body mass index
CCC	Canadian Consensus Criteria
CFS	Chronic fatigue syndrome
DSQ	DePaul Symptom Questionnaire
ME	Myalgic encephalomyelitis
ME/CFS	Myalgic encephalomyelitis/chronic fatigue syndrome
PEM	Post-exertional malaise
ROC	Receiver operating characteristic
SF-36	Medical Outcomes Study 36-item Short-Form Health Survey
VSURF	Variable selection using random forests
